# Rate of forcing and the forecastability of critical transitions

**DOI:** 10.1002/ece3.2531

**Published:** 2016-10-05

**Authors:** Christopher F. Clements, Arpat Ozgul

**Affiliations:** ^1^ Department of Evolutionary Biology and Environmental Studies University of Zurich Zurich Switzerland

**Keywords:** critical slowing down, early warning signals, population collapse, rate of forcing, regime shifts, tipping points

## Abstract

Critical transitions are qualitative changes of state that occur when a stochastic dynamical system is forced through a critical point. Many critical transitions are preceded by characteristic fluctuations that may serve as model‐independent early warning signals, implying that these events may be predictable in applications ranging from physics to biology. In nonbiological systems, the strength of such early warning signals has been shown partly to be determined by the speed at which the transition occurs. It is currently unknown whether biological systems, which are inherently high dimensional and typically display low signal‐to‐noise ratios, also exhibit this property, which would have important implications for how ecosystems are managed, particularly where the forces exerted on a system are anthropogenic. We examine whether the rate of forcing can alter the strength of early warning signals in (1) a model exhibiting a fold bifurcation where a state shift is driven by the harvesting of individuals, and (2) a model exhibiting a transcritical bifurcation where a state shift is driven by increased grazing pressure. These models predict that the rate of forcing can alter the detectability of early warning signals regardless of the underlying bifurcation the system exhibits, but that this result may be more pronounced in fold bifurcations. These findings have important implications for the management of biological populations, particularly harvested systems such as fisheries, and suggest that knowing the class of bifurcations a system will manifest may help discriminate between true‐positive and false‐positive signals.

## 1 Introduction

Critical transitions are sudden, large, often irreversible, and usually unwanted changes in the state of dynamical systems (Dakos et al., 2008; Scheffer et al., 2009). Such events are caused by increasing external forces, which decrease a system's resilience to perturbations (Scheffer, 2009). If the system is pushed hard enough, it may move away from its original stabilizing kinetics and pass through a tipping point, which can lead to abrupt changes (Scheffer, 2009). These tipping points are commonly associated with bifurcations in the associated mean field dynamics. Such dramatic shifts have been identified in a wide range of systems from lasers (Tredicce et al., 2004) to neurons (Matsumoto & Kunisawa, 1978). In ecology, such shifts include the sudden collapse of ecosystems (Benedetti‐Cecchi, Tamburello, Maggi, & Bulleri, 2015; Biggs, Carpenter, & Brock, 2009; Carpenter et al., 2011; Guttal & Jayaprakash, 2008; Seekell, Carpenter, Cline, & Pace, 2012) and population extinctions (Drake & Griffen, 2010). Significant recent effort has been invested in identifying statistical signatures preceding these tipping events (Biggs et al., 2009; Boettiger, Ross, & Hastings, 2013; Brock & Carpenter, 2012; Dakos, Kéfi, Rietkerk, van Nes, & Scheffer, 2011; Dakos et al., 2008, 2012; Folke et al., 2004; Scheffer et al., 2012) and has yielded a suit of approaches that can be used to detect “early warning signals” of an approaching tipping point (Dakos et al., 2012).

The pressures exerted on ecological systems take many different forms, such as climate‐derived alterations of environmental conditions (Foden et al., 2013; Thomas et al., 2004), overexploitation of commercially important species (Hutchings & Myers, 1994), and emerging infectious diseases (Bosch, Martínez‐Solano, & García‐París, 2001). The nature and intensity of these threats can significantly alter the speed at which a population declines toward extinction (Cardillo et al., 2005), with, for example, catastrophic collapses attributed to disease outbreaks (Bosch et al., 2001) or overfishing (Hutchings, 2000). These pressures have the potential to destabilize a system to the point that a critical threshold is crossed. Forecastability of such an event could allow intervention before a population or ecosystem collapses (Biggs et al., 2009; Carpenter et al., 2011; Drake & Griffen, 2010). Thus, reliable identification of early warning signals is important for conservation prioritization and management (Clements, Drake, Griffiths, & Ozgul, 2015).

**Figure 1:**
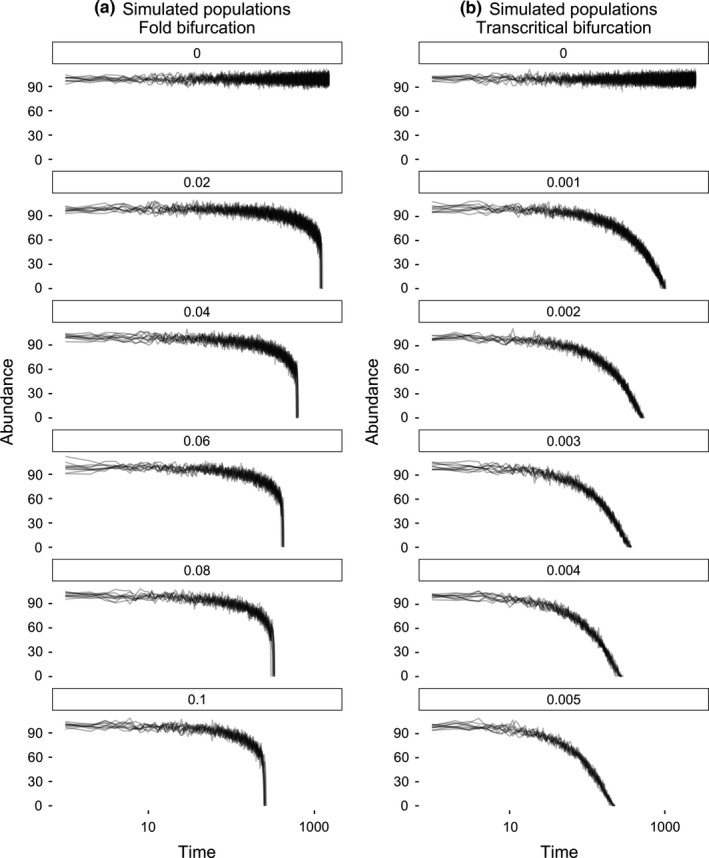
Simulated population collapses with different rates of change of the harvesting parameter (a subset of six rates of forcing are shown), using the (a) the fold bifurcation model described in Dakos et al. (2012) and (b) the transcritical bifurcation model described in Kéfi et al. (2013)

In nonbiological systems, the rate at which a system approaches a tipping point has been shown to alter not only the timing of a critical transition but also the behavior of the system in the vicinity of the critical point (Ashwin, Wieczorek, Vitolo, & Cox, 2012; Tredicce et al., 2004). Such changes in a system's short‐term behavior, driven by the rate of forcing, have the potential to affect the strength of generic leading indicators prior to a critical transition, for instance by reducing the time it takes a population to collapse so that the speed of forcing affects the amount of data available to detect early warning signals (Figure 1). Longer series of observations mean not only that more precise estimates of statistical properties (such as autocorrelation at first lag) can be made, but also that there is a greater period of time during which one can detect a system before it returns to equilibrium.

Alternatively, when the amount of data available to be collected is fixed (say from the start of historic sampling to the current day), rate of forcing could alter the strength of early warning signals by altering how destabilized a system becomes over a given time period. As early warning signals are based on the change in leading indicators across a given time series, it is the relative instability of a system with respect to conditions at start of the time series that is important for forecastability. Consequently, slow rates of forcing where time‐series length is invariant limit the scope for detecting change in a leading indicator, whereas faster rates of change over the same period can push a system further from equilibrium within the same amount of time. Understanding how the rate of forcing of biological systems affects the strength of early warning signals is thus important to their proper use for conservation intervention and planning.

Here we use models known to exhibit statistical signals of critical slowing down before to investigate how the rate of forcing of a system alters the strength of early warning signals of population collapse. Using two models, one exhibiting a fold bifurcation and the other a transcritical bifurcation, we simulate populations subject to a range of forcing speeds and show that the rate of forcing has the potential to alter the detectability of generic early warning signals in biological systems, both through the length of time series available to analyze and by directly affecting the dynamics of a system prior to a collapse.

## 2 Methods

### 2.1 Simulated time series

The first model describes a population that grows logistically and moves from an underexploited to an overexploited state via a fold catastrophe (Scheffer, 2009). This change is driven by an increase in the rate of harvesting of the population (*c*) (Dakos et al., 2012; May, 1977). The abundance of the population was simulated using the stochastic differential equation

$$dx\;=\;\left(rx\left(1-\frac{x}{K}\right)\;-\;c_{t}\frac{x^{2}}{x^{2}+h^{2}}\right)dt\;+\;\sigma xdW,$$

where *r* is the population's growth rate, *K* is the carrying capacity, *h* is the half‐saturation constant, and *c* is the harvesting rate. d*W* is a normally distributed (white noise process) stochastic perturbation with mean = 0 and σ = 0.5 that was applied at each time step with expectation μ(*x*_*t*_, *t*) and intensity σ(*x*_*t*_, *t*)^2^. The abundance simulations were implemented with a time step (δ*t*) of 0.025 using the Euler approximation.

The second model is modification of the fold bifurcation model above, where the population grows logistically and is forced through a transcritical bifurcation by overharvesting of individuals via a linear harvesting function (Kéfi, Dakos, Scheffer, Van Nes, & Rietkerk, 2013; Figure 1b). Thus, the abundance of a population was simulated using the stochastic differential equation

$$dx\;=\;\left(rx\left(1-\frac{x}{K}\right)\;-\;c_{t}x\right)dt\;+\;\sigma xdW.$$

Parameter values for both models are shown in Table 1.

**Table 1:** The parameter values for the fold bifurcation model (described in Dakos et al. 2012) and the transcritical model (described in Kéfi et al. 2013)

Parameter	Description	Units	Value
Fold bifurcation model			
*r*	Population growth rate	time^−1^	1^a^
*K*	Carrying capacity	–	100
*h*	Half‐saturation constant	–	1^a^
*c*	Harvesting rate	× time^−1^	Various
σ	Variance of white noise	–	0.5
Transcritical bifurcation model			
*r*	Population growth rate	time^−1^	1
*K*	Carrying capacity	–	100
*c*	Harvesting rate	× time^−1^	Various
σ	Variance of white noise	–	0.5

a Indicates parameter values taken from the relevant publications.

To model various rates of change of the system, we simulated 100 replicates of each of 11 different rates of change of the *c* parameters. In each of these 11 scenarios, the value of *c* increased linearly from 0 by a fixed factor at every whole time step. For the fold bifurcation model, those values were *c = *(0, 0.01, 0.02, …, 0.1), while for the transcritical bifurcation, those values were *c = *(0, 0.001, 0.0015, …, 0.005). These values were chosen so that simulated populations from the two models collapsed over similar time frames (Figure 1). These treatments gave a total of 1,100 simulated populations per model, which drove populations through critical transitions at varying points in time (Figure 1a).

### 2.2 Calculation of early warning signals

We calculate commonly applied generic leading indicators of population collapse using the “earlywarnings” package in R (Dakos et al., 2012). These generic leading indicators are based on statistical signals of a time series and change predictably as a population approaches a critical transition (Dakos et al., 2012). The indicators we tested were the autocorrelation at the first lag (similarity of value at time *t* to the value at *t* − 1), density ratio (ratio of low frequencies to high frequencies within a rolling window), first‐order autoregressive coefficient (ar1), return rate (rate of return to equilibrium after a perturbation), and the standard deviation of the abundance time series. A number of these leading indicators are theoretically highly correlated (for example, return rate is calculated as 1/ar1); however, especially with noisy data, these indicators have been shown to often perform differently (Clements et al., 2015). Following standard practice (Dakos et al., 2012), indicators were calculated within a predefined time window (typically 50% of the time series) using a sliding window through time after the time series had been detrended (Gaussian detrending for the fold bifurcation model and linear detrending for the transcritical bifurcation model). For all simulated populations, we calculated early warning signals across the full population time series until 10 time steps before the first count of less than one individual (the time the population collapsed). To summarize findings across model simulations, we present the Kendall's tau correlation coefficients of leading indicators prior to a collapse—strong positive correlations between time and an indicator indicate a transition is being approached for all leading indicators except return rate, where a strong negative correlation indicates an approaching transition (Dakos et al., 2008, 2012).

We present two analyses of the data. In the first analysis, all time series were standardized in length to the length of the shortest time series (~240 time points for each model). This was achieved by analyzing the final 240 time points prior to collapse for each population independently. Standardizing the time series removed any effect of time‐series length varying with the rate of forcing of the system (Figure 1). The second analysis used nonstandardized time‐series lengths, which were determined by the timing of collapse of the population. We debate the different real‐world scenarios each of these analyses represents in the discussion.

## 3 Results

### 3.1 Simulations

**Figure 2:**
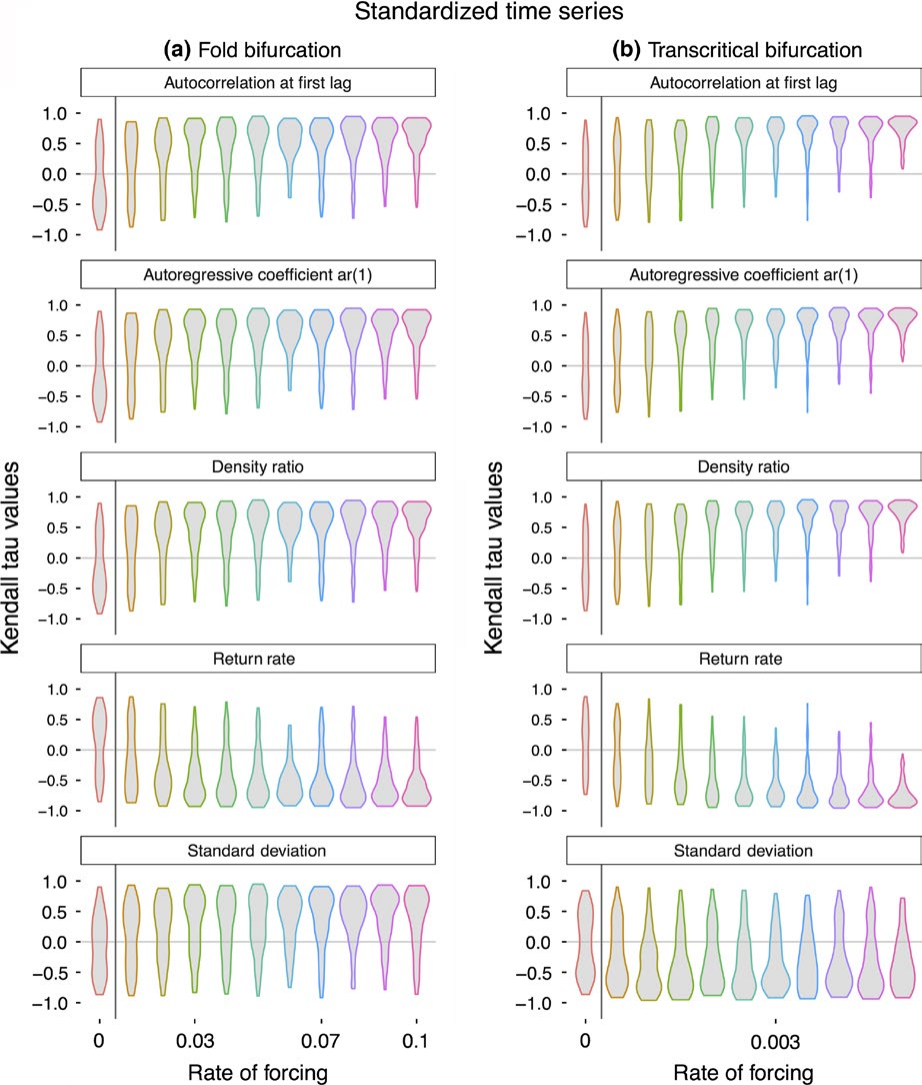
Strength of the trend in five leading indicators of population collapse when time‐series lengths were standardized (see section 2) across (a) simulated population collapses exhibiting a fold bifurcation, and (b) simulated population collapses exhibiting a transcritical bifurcation. Violin plots indicate the distribution of Kendall tau values for each rate of forcing

**Figure 3:**
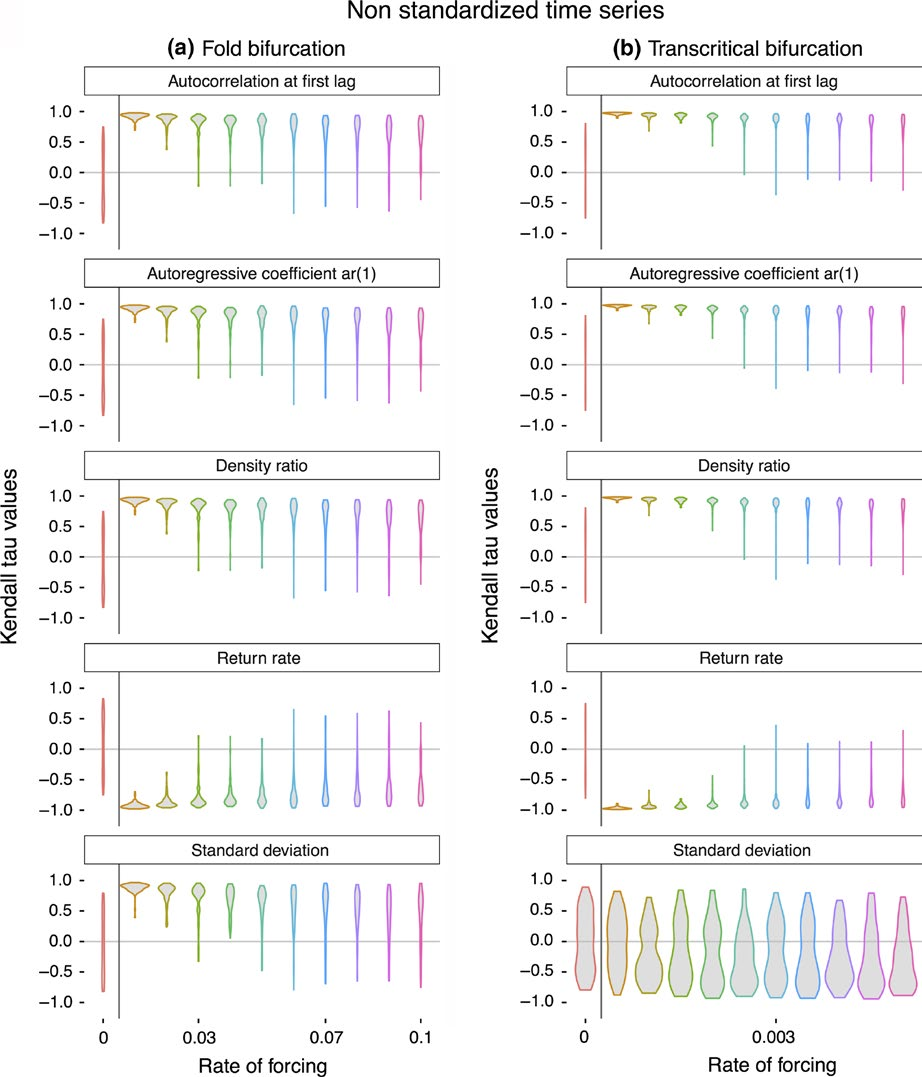
Strength of the trend in five leading indicators of population collapse when time‐series lengths were nonstandardized (see section 2) across (a) simulated population collapses exhibiting a fold bifurcation, and (b) simulated population collapses exhibiting a transcritical bifurcation. Violin plots indicate the distribution of Kendall tau values for each rate of forcing

In both models, the rate of system forcing altered the population dynamics and timing of extinction, and thus the length of the time series prior to collapse (Figure 1a, b). Because the rate of forcing altered the length of the time series available to analyze, we performed two analyses on the population data—one where the length of the time series was standardized to investigate whether rate of forcing alone alters the strength of early warning signals, and one where the time‐series length was allowed to vary with the rate of forcing to investigate the more realistic combined effects of both rate of forcing and time‐series length. When the length of the time series was standardized, the rate of forcing significantly altered the strength of early warning signals in both models, with faster rates of forcing producing stronger early warning signals (Figure 2). This pattern was reversed when time‐series length was allowed to vary, where slow rates of forcing produced longer time series which in turn led to stronger early warning signals (Figure 3a, Table 1). The one exception to this was in the transcritical bifurcation model, where the Kendall tau values of the standard deviation remained largely unaffected by the rate of forcing (a finding not altered by either Gaussian or linear detrending, results not shown).

Time‐series length also altered the distribution of Kendall tau values in the constant treatments, where theory suggests that there should be no detectable trend in the five leading indicators (Kendall tau values ≈ 0). While in both the standardized and nonstandardized time series, Kendall tau values were approximately uniformly distributed around 0, the variance was much larger in the standardized time series, suggesting it might be difficult disentangling false‐positive from true‐positive signals.

## 4 Discussion

The possibility of generic early warning signals gives hope that population collapse may be predicted prior to its occurrence, a potentially useful tool for conservation prioritization and management. Such approaches could be particularly applicable to harvested systems such as fisheries (Vasilakopoulos & Marshall, 2015), where high‐quality long‐term data are regularly collected, and the forces exerted upon a system are inherently manageable (Clements et al., 2015; Worm et al., 2006). Understanding how the rate of forcing of a system affects the rate of population collapse and the trends in generic leading indicators of stability, upon which the majority of early warning signals are based, is therefore critical for accurately inferring whether a population is at risk of extinction. Here, we demonstrate, using model simulations, that in general rate of forcing of a system has the potential to significantly alter the detectability of proposed generic early warning signals of population collapse. One possible exception to this appears to be the standard deviation of the time series, which in the transcritical bifurcation model seems unaffected by the rate of forcing, suggesting that there are some differences in the response of each system based on the nature of the underlying bifurcation. Both fold and transcritical transitions are expected to generate early warning signals prior to population collapse (Clements et al., 2015; Dakos et al., 2012; Drake & Griffen, 2010), but our results suggest that not all leading indicators may not respond equivalently to various rates of forcing.

The rate of forcing of the system altered the trends of four of the five generic leading indicators in the models, regardless of the whether the time‐series length was standardized (Figures 2a and 3a, Table 1), although this effect was much more pronounced when time‐series lengths were allowed to varying with the rate of forcing (Figure 2b). The more pronounced result in the nonstandardized time series is unsurprising, as longer time series necessarily contain more information of precollapse dynamics, and allowing for trends in leading indicators to be more easily identified. Of concern may be the fact that slow rates of forcing when the time series are standardized lead to large variance in the Kendall tau values of the five leading indicators tested, both in populations known to collapse, and also those which persist (rate of forcing = 0, Figure 2). This strongly suggests that disentangling false‐positive and true‐positive early warning signals are liable to be problematic in real‐world scenarios (Clements et al., 2015), and strengthens the case for the inclusion of alternative warning signals (such as those based on shifts in fitness‐related phenotypic traits) alongside the generic abundance‐based measures assessed here (Clements & Ozgul, 2016).

**Figure 4:**
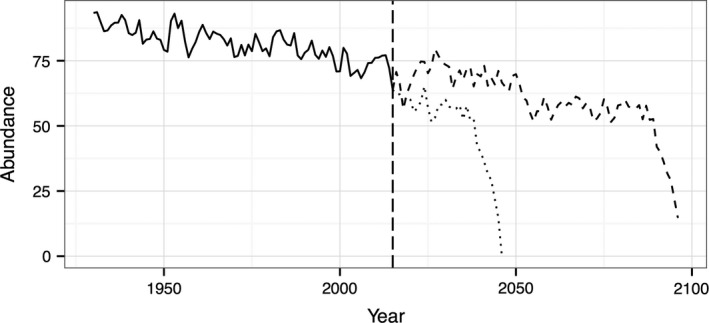
The length of time series for currently extant (in 2015, vertical dashed line) populations is determined by the monitoring effort in the preceding decades (solid black line) rather than the timing of the population crash. The total possible length of a time series is determined not only by the monitoring effort but also by the time to collapse, which in turn is governed by the rate of forcing of the system (dotted and dashed black lines)

The results presented here, using standardized time‐series lengths and lengths allowed to vary with the rate of forcing, mimic different potential real‐world scenarios. In the first instance, the length of the time series available to detect early warning signals is limited by the time that it takes for a population to collapse (Figures 1 and 3). This scenario is unlikely to represent currently available conservation data because if one is seeking to detect early warning signals of population collapse, the population of conservation interest will not have already crashed. Thus, when the population of interest is still viable, the length of the time series is necessarily bounded by the length of the monitoring period (Figure 4). If, however, one considers a population that has not yet crashed, and will continue to be monitored into the future, the potential length of the time series prior to a crash occurring is limited by not only the monitoring effort but also the time a crash occurs (Figure 4). Consequently, the results presented using standardized time‐series lengths are more pertinent to detecting early warning signals of collapse for use in current conservation prioritization, while the results presented where time‐series length is allowed to vary may be more analogous to data collected into the future.

In conclusion, we show that the predictability of population collapse is likely to be not only a function of the rate at which a system is forced away from stability, but may also depend on the underlying dynamics. A more complete understanding of a system may therefore be required before we are able to identify whether signals generated by currently available generic leading indicators are likely reliable.

## Conflict of Interest

None declared.

## References

[ece32531-bib-0001] Ashwin, P. , Wieczorek, S. , Vitolo, R. , & Cox, P. (2012). Tipping points in open systems: Bifurcation, noise‐induced and rate‐dependent examples in the climate system. Philosophical Transactions of the Royal Society A: Mathematical, Physical and Engineering Sciences, 370, 1166–1184. 10.1098/rsta.2011.0306 22291228

[ece32531-bib-0002] Benedetti‐Cecchi, L. , Tamburello, L. , Maggi, E. , & Bulleri, F. (2015). Experimental perturbations modify the performance of early warning indicators of regime shift. Current Biology, 25, 1867–1872. 26166776 10.1016/j.cub.2015.05.035

[ece32531-bib-0003] Biggs, R. , Carpenter, S. R. , & Brock, W. A. (2009). Turning back from the brink: Detecting an impending regime shift in time to avert it. Proceedings of the National Academy of Sciences of the United States of America, 106, 826–831. 19124774 10.1073/pnas.0811729106 PMC2630060

[ece32531-bib-0005] Boettiger, C. , Ross, N. , & Hastings, A. (2013). Early warning signals: The charted and uncharted territories. Theoretical Ecology, 6, 255–264.

[ece32531-bib-0006] Bosch, J. , Martínez‐Solano, I. , & García‐París, M. (2001). Evidence of a chytrid fungus infection involved in the decline of the common midwife toad (*Alytes obstetricans*) in protected areas of central Spain. Biological Conservation, 97, 331–337.

[ece32531-bib-0007] Brock, W. A. , & Carpenter, S. R. (2012). Early warnings of regime shift when the ecosystem structure is unknown. PLoS One, 7, e45586. 23029118 10.1371/journal.pone.0045586 PMC3448650

[ece32531-bib-0008] Cardillo, M. , Mace, G. M. , Jones, K. E. , Bielby, J. , Bininda‐Emonds, O. R. P. , Sechrest, W. , … Purvis, A. (2005). Multiple causes of high extinction risk in large mammal species. Science, 309, 1239–1241. 16037416 10.1126/science.1116030

[ece32531-bib-0010] Carpenter, S. R. , Cole, J. J. , Pace, M. L. , Batt, R. , Brock, W. A. , Cline, T. , … Weidel, B. (2011). Early warnings of regime shifts: A whole‐ecosystem experiment. Science, 332, 1079–1082. 21527677 10.1126/science.1203672

[ece32531-bib-0011] Clements, C. F. , Drake, J. M. , Griffiths, J. I. , & Ozgul, A. (2015). Factors influencing the detectability of early warning signals of population collapse. The American Naturalist, 186, 50–58. 10.1086/681573 26098338

[ece32531-bib-0012] Clements, C. F. , & Ozgul, A. (2016). Including trait‐based early warning signals helps predict population collapse. Nature Communications, 7, 10984. 10.1038/ncomms10984 PMC4820807 27009968

[ece32531-bib-0014] Dakos, V. , Carpenter, S. R. , Brock, W. A. , Ellison, A. M. , Guttal, V. , Ives, A. R. , … Scheffer, M. (2012). Methods for detecting early warnings of critical transitions in time series illustrated using simulated ecological data. PLoS One, 7, e41010. 22815897 10.1371/journal.pone.0041010 PMC3398887

[ece32531-bib-0015] Dakos, V. , Kéfi, S. , Rietkerk, M. , van Nes, E. H. , & Scheffer, M. (2011). Slowing down in spatially patterned ecosystems at the brink of collapse. The American Naturalist, 177, E153–E166. 10.1086/659945 21597246

[ece32531-bib-0016] Dakos, V. , Scheffer, M. , van Nes, E. H. , Brovkin, V. , Petoukhov, V. , & Held, H. (2008). Slowing down as an early warning signal for abrupt climate change. Proceedings of the National Academy of Sciences of the United States of America, 105, 14308–14312. 18787119 10.1073/pnas.0802430105 PMC2567225

[ece32531-bib-0017] Drake, J. , & Griffen, B. (2010). Early warning signals of extinction in deteriorating environments. Nature, 467, 456–459. 20827269 10.1038/nature09389

[ece32531-bib-0019] Foden, W. B. , Butchart, S. H. M. , Stuart, S. N. , Vié, J.‐C. , Akçakaya, H. R. , Angulo, A. , … Mace, G. M. (2013). Identifying the world's most climate change vulnerable species: A systematic trait‐based assessment of all birds, amphibians and corals (ed S Lavergne). PLoS One, 8, e65427. 23950785 10.1371/journal.pone.0065427 PMC3680427

[ece32531-bib-0020] Folke, C. , Carpenter, S. , Walker, B. , Scheffer, M. , Elmqvist, T. , Gunderson, L. , & Holling, C. S. (2004). Regime shifts, resilience, and biodiversity in ecosystem management. Annual Review of Ecology, Evolution, and Systematics, 35, 557–581.

[ece32531-bib-0021] Guttal, V. , & Jayaprakash, C. (2008). Changing skewness: An early warning signal of regime shifts in ecosystems. Ecology Letters, 11, 450–460. 18279354 10.1111/j.1461-0248.2008.01160.x

[ece32531-bib-0022] Hutchings, J. A. (2000). Collapse and recovery of marine fishes. Nature, 406, 882–885. 10972288 10.1038/35022565

[ece32531-bib-0023] Hutchings, J. A. , & Myers, R. A. (1994). What can be learned from the collapse of a renewable resource? Atlantic cod, *Gadus morhua*, of newfoundland and labrador. Canadian Journal of Fisheries and Aquatic Sciences, 51, 2126–2146.

[ece32531-bib-0024] Kéfi, S. , Dakos, V. , Scheffer, M. , Van Nes, E. H. , & Rietkerk, M. (2013). Early warning signals also precede non‐catastrophic transitions. Oikos, 122, 641–648.

[ece32531-bib-0025] Matsumoto, G. , & Kunisawa, T. (1978). Critical slowing‐down near the transition region from the resting to time‐ordered states in squid giant axons. Journal of the Physical Society of Japan, 44, 1047–1048.

[ece32531-bib-0026] May, R. M. (1977). Thresholds and breakpoints in ecosystems with a multiplicity of stable states. Nature, 269, 471–477.

[ece32531-bib-0027] Scheffer, M. (2009). Alternative stable states In Levin S. A., & Strogatz S. H. (Eds.), Critical transitions in nature and society (pp. 11–36). Princeton: Princeton University Press.

[ece32531-bib-0028] Scheffer, M. , Bascompte, J. , Brock, W. A. , Brovkin, V. , Carpenter, S. R. , Dakos, V. , … Sugihara, G. (2009). Early‐warning signals for critical transitions. Nature, 461, 53–59. 19727193 10.1038/nature08227

[ece32531-bib-0029] Scheffer, M. , Carpenter, S. R. , Lenton, T. M. , Bascompte, J. , Brock, W. , Dakos, V. , … Vandermeer, J. (2012). Anticipating critical transitions. Science, 338, 344–348. 23087241 10.1126/science.1225244

[ece32531-bib-0030] Seekell, D. A. , Carpenter, S. R. , Cline, T. J. , & Pace, M. L. (2012). Conditional heteroskedasticity forecasts regime shift in a whole‐ecosystem experiment. Ecosystems, 15, 741–747.

[ece32531-bib-0031] Thomas, C. D. , Cameron, A. , Green, R. E. , Bakkenes, M. , Beaumont, L. J. , Collingham, Y. C. , … Williams, S. E. (2004). Extinction risk from climate change. Nature, 427, 145–148. 14712274 10.1038/nature02121

[ece32531-bib-0032] Tredicce, J. R. , Lippi, G. L. , Mandel, P. , Charasse, B. , Chevalier, A. , & Picqué, B. (2004). Critical slowing down at a bifurcation. American Journal of Physics, 72, 799.

[ece32531-bib-0033] Vasilakopoulos, P. , & Marshall, C. T. (2015). Resilience and tipping points of an exploited fish population over six decades. Global Change Biology, 21, 1834–1847. 25545249 10.1111/gcb.12845

[ece32531-bib-0034] Worm, B. , Barbier, E. B. , Beaumont, N. , Duffy, J. E. , Folke, C. , Halpern, B. S. , … Watson, R. (2006). Impacts of biodiversity loss on ocean ecosystem services. Science, 314, 787–790. 17082450 10.1126/science.1132294

